# Tapered fiber probe-based optical cardiac pacemaker

**DOI:** 10.3389/fbioe.2025.1675219

**Published:** 2025-11-11

**Authors:** Yanzheng Xie, Xiaoshuai Liu

**Affiliations:** 1 School of Traditional Chinese Medicine, Jiangsu Medical College, Yancheng, China; 2 School of Physics and Materials Science, Guangzhou University, Guangzhou, China

**Keywords:** biophotonics, tapered fiber probe, cardiac pacemaker, arrhythmia disease, optical manipulation

## Abstract

**Introduction:**

The disorders of cardiac rhythm usually induce severe cardiovascular pathologies which might pose significant threats to human health. Although multiple cardiac pacing modalities have been developed, most of them face potential limitations including structural complexity, spatiotemporal imprecision and invasive implantation requirements, thereby constraining their widespread biomedical applications.

**Methods:**

By leveraging the unique long-range focusing capability, we establish a tapered-fiber-probe (TFP) strategy enabling non-contact and highprecision near-infrared (NIR) optical pacing in zebrafish embryos, where sustained micron-scale spatial precision (3 μm FWHM) was achieved at physiologically relevant working distances.

**Results:**

Systematic characterizations revealed developmental-stage-dependent pacing sensitivity, with pacing efficacy progressively decreasing during early cardiogenesis (36 dpf) and stabilizing upon cardiac maturation (≥6 dpf). Meanwhile, anatomical mapping demonstrated 1.7-fold greater photosensitivity in sinoatrial regions compared to ventricular myocardium. Calcium imaging confirmed a photothermal mechanism wherein optical absorption of irradiated myocardial tissue activates thermosensitive protein channels, triggering calcium ion influx and subsequent depolarization.

**Discussion:**

The proposed strategy enables spatiotemporally precise cardiac conduction and establishes a proof-of-concept platform for non-contact optical pacing in zebrafish embryos, which might provide potential bio-optical tool development for basic arrhythmia research *in vivo*.

## 1 Introduction

Cardiac arrhythmias represent a series of life-threatening disorders involving over 60 million people globally, characterized by abnormal electrical conduction patterns that disrupt synchronized myocardial contraction (Grune et al., 2021; Lazzerini et al., 2022; Sayers et al., 2025; Schwartz et al., 2020). Conventional implantable electronic pacemakers (Choi et al., 2021), while clinically indispensable, face fundamental limitations including invasive surgical implantation risks, finite battery lifespan requiring replacement surgeries (Ouyang et al., 2019), electromagnetic interference with MRI diagnostics (Vardas, 2024), and limited spatial resolution (>1 mm) that precludes localized conduction pathway modulation (Prominski et al., 2022; Wang E. et al., 2025). These constraints have motivated the exploration of alternative pacing modalities, among which optical techniques have emerged as promising candidates due to their inherent non-invasiveness, compatibility with functional imaging, and cellular-scale spatial precision (Lee et al., 2020; Naimovičius et al., 2025; Zhou et al., 2025).

Optical pacing leverages photonic energy to modulate cardiac electrophysiology through multiple biophysical mechanisms. Optogenetic approaches utilize genetically encoded light-sensitive ion channels to achieve millisecond-precision depolarization (Entcheva and Kay, 2021; Joshi et al., 2020), yet require viral transfection with associated immunogenicity risks (Li et al., 2024). Moreover, the complicated genetic manipulation might alter native cardiomyocyte electrophysiology. Consequently, non-genetic near-infrared (NIR) optical pacing (700–1100 nm) has emerged as a promising alternative strategy (Liu et al., 2021), which depends on light delivery systems capable of achieving cellular-scale spatial confinement while maintaining sufficient energy density for electrophysiological modulation (Parameswaran et al., 2019). This mechanism circumvents genetic modification requirements while achieving stimulation precision determined by the optical confinement volume (Ronchi et al., 2024). Especially, inspired by fiber-optic endoscopy techniques (Wen et al., 2023; Wu G. et al., 2024), attempts have been made to leverage optical fibers for delivering near-infrared laser beam to deep cardiac tissue, achieving real-time cardiac pacing to modulate heart rate *in vivo* (Chung and Chung, 2019). However, conventional single-mode fibers (SMFs) exhibit significant beam divergence that compromises spatial precision (Avsievich et al., 2020; Xie and Liu, 2022). By contrast, tapered fiber probes (TFPs) represent an engineered solution that combines waveguide confinement with distal beam shaping, generating micro-scale focal spots at an enhanced working distances through controlled core diameter design (Singh et al., 2023; Wang et al., 2022; Zhang et al., 2023). This unique beam propagation characteristic enables unprecedented spatial precision in cardiac stimulation, thus holding great promise to enable optical pacing in a contactless and high-precision manner (Yu et al., 2020).

In this study, by integrating an elaborately designed TFP, we introduce an innovative optical cardiac pacemaker for the real-time control of heart rate in zebrafish hearts. By leveraging the TFP’s unique long-range focusing capability, sustained micron-scale spatial precision was achieved at physiologically relevant working distances. Through systematic exploration in developing zebrafish embryos, we quantify developmental-stage-dependent pacing sensitivity across 3–8 days post-fertilization (dpf), map anatomical variation in pacing thresholds across key cardiac regions and then demonstrate spatiotemporal control of cardiac rhythm with cellular resolution. Furthermore, the inherent physiology mechanism was explored via simultaneous calcium imaging.

## 2 Methods

### 2.1 Sample preparation of zebrafish embryo

Fertilization was achieved through natural spawning, with embryos maintained at 28 °C in saltwater supplemented with 0.003% w/v phenylthiourea to inhibit pigment synthesis. For optical pacing experiments, larvae were transferred to 15 × 15 mm cover glasses using a micropipette, positioned via a fine hair loop to orient the body and tail against the glass surface for optimal optical access, and immobilized in 2% agarose gel to prevent movement during stimulation protocols. All procedures strictly adhered to the animal welfare guidelines of Jiangsu Vocational College of Medicine.

### 2.2 Fabrication of TFP

The used TFP was fabricated from commercial single-mode optical fibers (Corning Inc.; FC/PC connector, core diameter: 9 μm, cladding diameter: 125 μm) using a flame-heating technique. The fiber buffer and polymer jacket were first removed with a precision stripper, after which the fiber was sheathed within a borosilicate glass capillary (inner diameter: 0.9 ± 0.05 mm, wall thickness: 0.1 ± 0.02 mm, length: 120 mm) to prevent mechanical deformation during processing. Under continuous flame heating for 60 s, the fiber was drawn axially at 2 mm/s, achieving a controlled diameter reduction from 125 μm to 10 μm over a 2.0 mm taper region. Finally, the pulling velocity was improved to be 10 mm/s within 0.1 s, and then the bare fiber was broken with a desired probe tip. Notably, this process yielded >90% geometric consistency across 20 independently fabricated probes, which enables high-throughput fabrication of identical TFPs with negligible experimental artifacts. Besides, all cardiac stimulation data presented in this study were acquired using a single, characterized tapered fiber probe (TFP) to eliminate inter-probe variability. This ensured consistent optical focusing performance across all experiments, including regional pacing sensitivity mapping and mechanistic investigations.

### 2.3 Cardiac rhythm characterization

Cardiac rhythm quantification was performed using a high-speed complementary metal-oxide-semiconductor camera (IDS Imaging Development Systems GmbH, Germany) operating at 15 fps. Real-time image acquisition was achieved with uncompressed video streams recorded on the computer directly. For motion trajectory analysis, time-lapse image sequences were processed in ImageJ using the semi-automated Manuel Tracking plugin, under which the point-to-point tracks can be realized to quantify the detailed motion trajectory for the heart in a real-time manner.

### 2.4 Cardiac cell culture

Cardiomyocytes were commercially obtained from HELP StemCell Innovations Ltd. (Nanjing, China). Cells were seeded at a density of 1 × 10^6^ cells per well in six-well plates pre-coated with 1% fibronectin and maintained in a humidified incubator (37 °C, 5% CO_2_) for 72 h. Following initial culture, the plating medium was replaced with cardiomyocyte maintenance medium. After 18 days of culture, cells were dissociated using cardiomyocyte dissociation solution, resuspended in fresh maintenance medium, and replated onto 1% fibronectin-coated 35 mm dishes. Experimental procedures were initiated 5–6 days post-replating, when cells exhibited stable contractile phenotypes confirmed by spontaneous beating synchrony and morphological maturity.

### 2.5 Calcium imaging

For calcium imaging experiments, cardiomyocytes were cultured in 35 mm confocal dishes. Prior to imaging, the maintenance medium was aspirated, and the cells were gently washed with Margo-Ring solution (130 mM NaCl, 1 mM MgCl_2_, 5 mM KCl, 10 mM glucose, 20 mM HEPES, and 2.5 mM CaCl_2_, pH 7.4). Subsequently, 2 mL of Margo-Ring solution containing 2 μM Fluo-4 AM dye was added, and the cells were incubated for 30 min at 37 °C in the dark. Following incubation, the cells were washed twice with Margo-Ring solution to thoroughly remove unbound dye. Fluorescence imaging was performed using a confocal microscope with an excitation wavelength of 488 nm, and high-resolution single-channel images were acquired.

### 2.6 Numerical simulation of light field distribution

The numerical simulations were performed by the finite element method using COMSOL Multiphysics 6.0 with the radio frequency module (electromagnetic wave, frequency domain) and perfectly matched layer boundary conditions. The incident light directed into TFP was set as a Gaussian beam with a wavelength of 1064 nm. The mesh sizes of the regions of the TFP, tissue and water were set as 100, 50 and 80 nm, respectively. The refractive indices of the fiber probe, tissue and water were set as 1.44, 1.39 and 1.33, respectively.

### 2.7 Statistical analysis

The statistical analysis was performed using GraphPad Prism (v8.0, GraphPad Software, Inc.). Results are presented as mean ± standard deviation (SD). For each embryo, heart rate data were quantified by averaging the heart rate over a 5-s period, which was selected to capture stable and reproducible cardiac responses as well as minimize the transient artifacts. The reported sample size (*n* = 5 or 8) refers to biological replicates (*i.e.*, independent zebrafish embryos or cardiac cells), ensuring conclusions reflect inter-individual variability. The sample size aligns with established standards in zebrafish cardiac modulation studies (Tang et al., 2020; Zhou et al., 2022). Comparison between two groups was performed by two-tailed *t*-test with Welch correction, assuming Gaussian distribution. Comparisons among multiple groups were performed by one-way ANOVA with Bonferroni correction. ns, no significant; ^*^, *P* < 0.05; ^**^, *P* < 0.01; ^***^, *P* < 0.001; ^****^, *P* < 0.0001. A *P* < 0.05 was considered to be statistically significant.

## 3 Results

### 3.1 Schematic illustration and experiment setup

**FIGURE 1:**
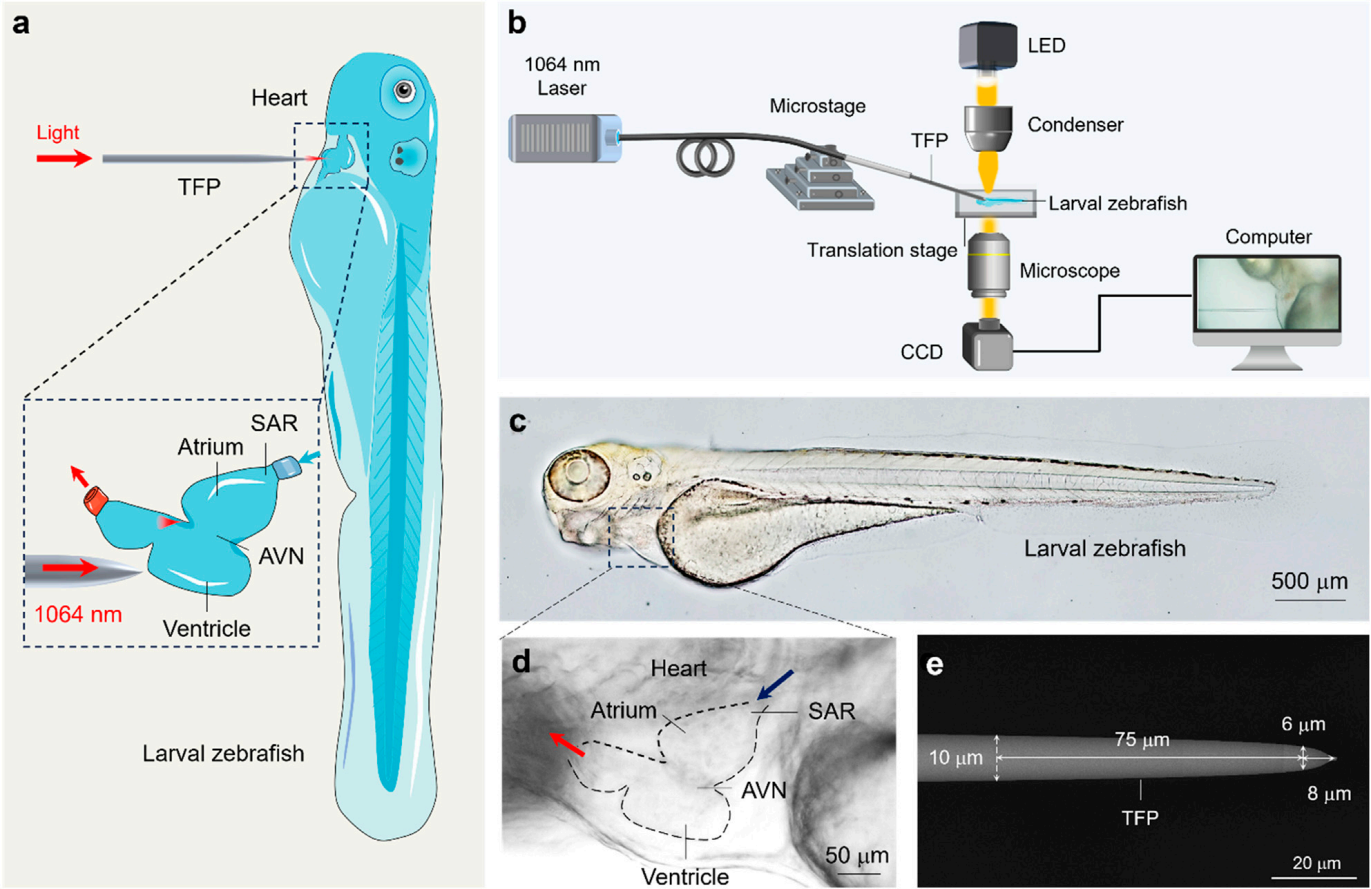
Experimental principles and material characterization. **(a)** Schematic illustration of experimental principle. The inset shows the optical stimulation of the zebrafish ventricle using the TFP. **(b)** Schematic illustration of experimental setup. **(c,d)** Optical micrograph of the zebrafish embryo **(c)** and the anatomy structure of zebrafish heart **(d)**. **(e)** SEM image of the fabricated TFP with annotated geometric dimensions.

Figure 1a illustrates the schematic diagram of optical cardiac pacemaker by using TFP. First, the TFP is positioned near the heart of zebrafish embryo, and its tip is precisely aligned with the target cardiac area. Concurrently, the NIR light beam is injected into the fiber probe, and meanwhile, an immediate acceleration was observed for the zebrafish heartbeat frequency. Notably, the magnitude of heartbeat acceleration can be dynamically modulated by adjusting the incident laser power, thereby achieving a real-time and precise control over the cardiac rhythm. Upon termination of light irradiation, the heartbeat returns to its intrinsic rhythm within seconds. Crucially, as the TFP emits micro-scale light focusing, systematic displacement of the probe facilitates quantitative characterization of the specific heart rate response elicited by stimulating distinct cardiac regions. This methodology provides a promising foundation for developing a novel methodology enabling non-contact, high-precision and programmable spatiotemporal control of cardiac pacing. Figure 1b illustrates the schematic of the experimental setup exploited in this study. A 6-day post-fertilization (dpf) zebrafish embryo is immobilized on the microscope stage using agarose to prevent undesired spatial displacement. The stage enables two-dimensional planar movement (manipulation precision: 1 µm) in the horizontal and vertical directions, allowing the heart of zebrafish embryo to be centered within the view field of microscope. Meanwhile, the TFP, fabricated using a flame-heating technique, is mounted on a six-axis micromanipulator. This enables three-dimensional control over its spatial position and inclination angle (positioning resolution: 500 nm). The proximal end of the fiber probe is connected to a near-infrared fiber laser operating in continuous-wave mode while at a wavelength of 1064 nm, which was chosen due to its minimal scattering by biological tissues (Liu et al., 2022), facilitating a greater penetration depth (Liu et al., 2024). Subsequently, the distal tip of the fiber probe is then maneuvered into proximity with the target cardiac region on the immobilized embryo. Furthermore, illumination light emitted from an overhead LED is concentrated via a condenser and then directed onto the zebrafish embryo to ensure optimal bright-field imaging. The whole experimental process is imaged through an inverted microscope objective beneath the sample, which transmits the signal to a computer-coupled CCD camera for rapid image capture and real-time video recording.

Notably, the zebrafish was chosen due to its complete optical transparency (Patton et al., 2021), benefiting from which it enables clear microscopic visualization of internal organs (Figure 1c). Specifically, it shares human-like cardiac conduction system (Bowley et al., 2022), including the atrium, ventricle, sinoatrial node (SAN, the primary pacemaker) and atrioventricular node (AVN, responsible for signal conduction delay), which are readily observable under standard brightfield microscopy (Figure 1d). This inherent transparency also facilitates real-time quantification of cardiac contraction dynamics. Crucially, zebrafish serve as an outstanding model organism for cardiovascular research due to their high degree of genetic homology with humans (>70% ortholog for disease-related genes) and recapitulation of fundamental cardiac electrophysiology during early development (González-Rosa, 2022). This physiological parallelism renders them an ideal biophotonic interface for investigating the fundamental mechanisms underlying TFP-mediated regulation of cardiac rhythm.

To enable the precise optical pacing with a micron level, a specially designed fiber probe was fabricated. As shown in Figure 1e, the probe geometry features a precisely controlled linear taper: the fiber diameter decreases from the standard 125 μm–10 μm over an initial 2 mm axial length. Subsequently, a secondary steeper linear taper reduces the diameter further from 10 μm to 6 μm within a constrained 75 μm segment. This design culminates in a well-defined 8 μm-long conical terminal tip, optimized for micron-scale spatial targeting during optical pacing.

### 3.2 Heart rate control by optical pacing with TFP

**FIGURE 2:**
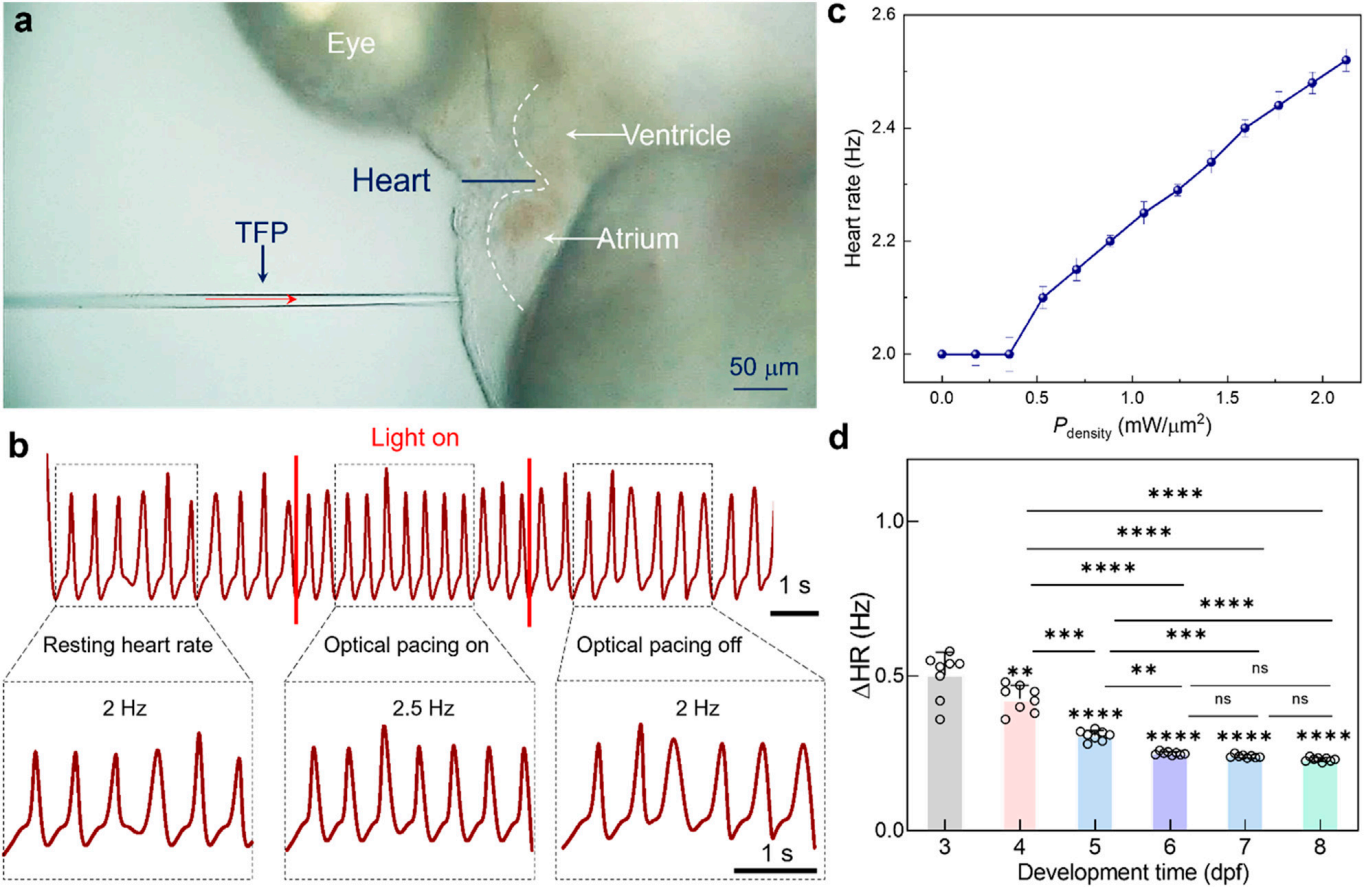
Optical pacing of zebrafish heart by using the laser beam delivered by TFP. **(a)** Brightfield micrograph illustrating cardiac pacing progress via focused light beam emission from TFP. **(b)** Cardiac rhythm traces before (baseline), during (stimulus), and after (recovery) optical pacing. **(c)** The calculated heart rate modulation as a function of laser power density (*n* = 5 larval zebrafish). **(d)** The calculated ΔHR as a function of developmental stage. Results are presented as mean ± SD (*n* = 8 larval zebrafish). Statistical comparison was analyzed by one-way ANOVA with Bonferroni correction. Significant differences relative to 3 dpf are indicated directly above the corresponding bars. ns, not significant. ns, no significant; ^**^ *P* < 0.01; ^***^ *P* < 0.001; ^****^ *P* < 0.0001.

To demonstrate the above design mechanism, a series of experiments were performed to validate the regulatory effect of the proposed TFP on zebrafish heart rate. Figure 2a presents the key experimental configuration: the TFP was precisely positioned to deliver 1064 nm laser irradiation to the atrium of zebrafish embryo, while cardiac dynamics were recorded simultaneously. Notably, the measured beam diameter at this working distance exhibited a full-width half-maximum (FWHM) of 6 μm (Supplementary Figure S1). As shown in Figure 2b, under resting conditions (pre-stimulation), the intrinsic heart rate was RHR = 2 Hz. Upon initiation of optical pacing, the heart rate immediately increased to 2.5 Hz and remained stable throughout the stimulation period. After that, the cessation of light irradiation triggered rapid restoration of the basal heart rate (*i.e.*, 2.0 Hz) within seconds. Critically, upon the removal of laser irradiation, the heart rate correspondingly returns to its pre-stimulation rate (Supplementary Movie S1). This reversible response conclusively demonstrates that the observed heartbeat acceleration is directly attributable to the optical stimulus rather than spontaneous cardiac behavior. Furthermore, the quantified dependence was characterized for the modulation flexibility on light power density (*P*_density_), which can be calculated based on laser power (*P*) and beam profile (A), *i.e.*, *P*_density_ = *P*/*A* = *P*/(π × (FWHM/2)^2^). As indicated in Figure 2c, irradiation at power levels below 0.53 mW/μm^2^ produced no discernible change in heart rate, *i.e.*, maintaining the resting hear rate of 2.0 Hz. Once the power density threshold of 0.53 mW/μm^2^ was exceeded, the heart rate increased linearly with laser power density, directly demonstrating the precise control of heart beating could be achieved by a real-time modulation of the laser power density.

Building upon these findings, the developmental-stage depended cardiac response was investigated for the zebrafish embryo to NIR optical pacing. As shown in Figure 2d, optical pacing was performed onto the larval zebrafish ranging from 3 to 8 dpf. Notably, the TFP was positioned to maintain consistent anatomical targeting relative to the heart across all stages. To quantitatively assess pacing efficacy, the heart rate change (ΔHR), defined as the difference between the stimulated heart rate and the intrinsic resting heart rate, was quantified across each developmental stage. Under consistent optical pacing parameters (*P*_*density*_ = 1.06 mW/μm^2^), the *Δ*HR delineates a critical developmental transition, *i.e.*, the shift from plasticity-driven variable sensitivity (3-6 dpf) to standardized excitable tissue response (≥6 dpf). In detail, the magnitude of *Δ*HR was measured to be 0.5 Hz, 0.4 Hz, 0.3 Hz, and 0.25 Hz respectively for 3, 4, 5, and 6-dpf embryos, thus indicating a progressive decrease in pacing sensitivity with development maturity. This attenuation likely reflects dynamic maturation of cardiac function and neural circuitry during early embryogenesis, thereby generating differentiate responsiveness to extrinsic stimulation. Nevertheless, for 6-8 dpf embryos, *Δ*HR plateaued at approximately 0.25 Hz, demonstrating stabilization of cardiac electrophysiological function. This functional homeostasis coincides with completed cardiac morphogenesis and autonomic nervous system maturation by 6 dpf, establishing uniform electrophysiological responsiveness to optical pacing stimuli delivered by TFP.

### 3.3 Cardiac region-targeted optical pacing using TFP

**FIGURE 3:**
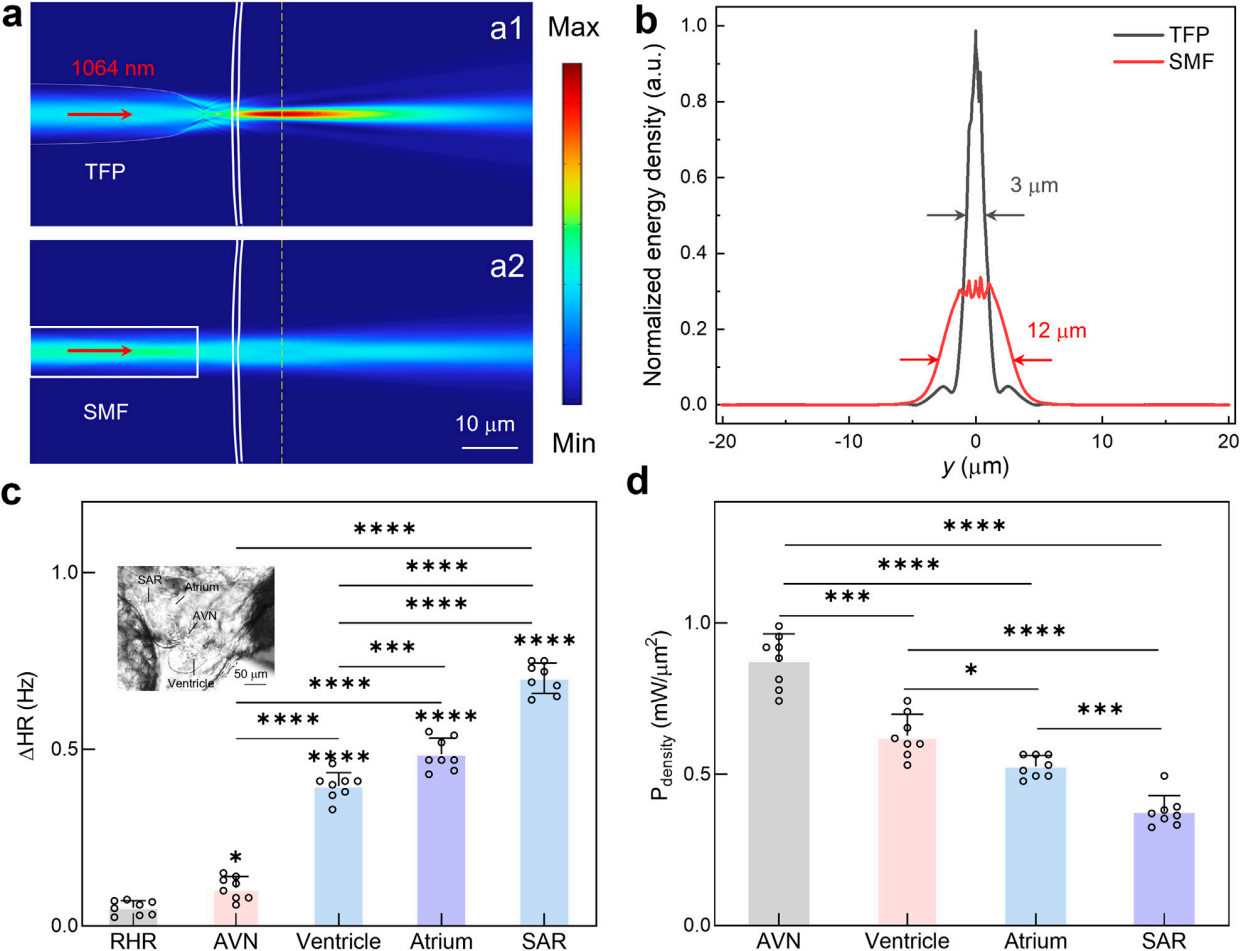
Cardiac region-targeted optical pacing using TFP. **(a)** Comparative analysis of simulated light field distribution emitted from TFP and SMF. **(b)** Normalized energy density along the yellow dashed line in a for both fiber types. **(c)** The calculated ΔHR during optical pacing of distinct cardiac regions. Results are presented as mean ± SD (*n* = 8 larval zebrafish). Statistical comparison was analyzed by one-way ANOVA with Bonferroni correction. Significant differences relative to RHR are indicated directly above the corresponding bars. **(d)** The required minimum optical power for successful pacing at different anatomical sites. Results are presented as mean ± SD (*n* = 8 larval zebrafish). Statistical comparison was analyzed by one-way ANOVA with Bonferroni correction. ns, not significant; ^*^ *P* < 0.05; ^**^ *P* < 0.01; ^***^ *P* < 0.001; ^****^ *P* < 0.0001.

Significantly, the light emission from TFP achieves micro-scale optical confinement, enabling targeted optical pacing at single-cell resolution within specific cardiac subregions. To validate this capability, a comparative analysis was conducted for the output light field distribution between the TFP and SMF (core diameter: 9 μm). As shown in Figure 3a, a significant remote focusing effect was observed for the proposed TFP, thus concentrating optical energy to precisely stimulate cardiomyocytes at the intended target site. Conversely, the SMF exhibits substantial beam divergence, resulting in broad-field near-infrared irradiation of myocardial tissue and consequently limited spatial precision for pacing applications. Furthermore, the axial energy density distributions were assessed quantitatively along the direction of propagation (*i.e.*, the yellow dashed line in Figure 3a). Notably, the proposed TFP demonstrates superior optical confinement compared to the SMF, *i.e.*, the FWHM was reduced from 12 μm to 3 μm, while the normalized peak energy density increased threefold compared to the SMF (Figure 3b). These findings confirm that substituting conventional SMF with the prosed TFP enables efficient optical field focusing. Benefited from this, it facilitates spatially precise stimulation of target cardiomyocytes at low-power stimulation, thus holding great promise to reduce pacing thresholds and enhance spatial resolution of cardiac electrophysiological modulation.

Leveraging the highly light focus effect of TFP, the region-specific responsiveness was systematically investigated for the zebrafish heart to near-infrared optical pacing. As depicted in Figure 3c, key cardiac structures, including the AVN, ventricle, atrium, and SAR, were sequentially targeted through precise spatial positioning of the TFP. Under identical optical stimulation parameters (*P*_density_ = 2.12 mW/μm^2^), these regions exhibited markedly distinct responses, with the measured *Δ*HR of 0.1 Hz, 0.4 Hz, 0.5 Hz, and 0.7 Hz for optical pacing at AVN, ventricle, atrium and SAR, respectively. These results demonstrate significantly higher sensitivity to optical pacing in the atrium compared to the ventricle or AVN, while the SAR, located in the right-inferior quadrant of the atrium near the venous inflow tract, exhibiting the most pronounced response.

To further validate these findings, the optical pacing thresholds, defined as the minimum incident power required to successfully initiate pacing, were quantified for different cardiac regions (Figure 3d). The results show the pacing thresholds differ substantially across cardiac regions, with a magnitude of 0.88, 0.60, 0.53 and 0.35 mW/μm^2^ for AVN, ventricle, atrium, and SAR, respectively. This threshold gradient, *i.e.*, progressively decreasing from outflow tract structures (AVN/ventricle) to the inflow tract pacemaker region (atrium/SAR), confirms the superior photosensitivity of sinoatrial nodal cardiomyocyte, which enables lower pacing thresholds as validated by region-specific stimulation mapping. Collectively, this spatial mapping reveals a critical functional hierarchy: the endogenous pacemaker complex (*i.e.*, SAR) exhibits approximately 1.7-fold greater photosensitivity than ventricular myocardium. This intrinsic differential sensitivity, combined with the micro-scale spatial precision afforded by the TFP, establishes a powerful methodology for efficient cardiac pacing. By strategically targeting the SAR with focused NIR light from TFP, it might achieve maximal chronotropic control at minimal power, thus establishing a potential foundation for advancing non-contact and precise optical pacing methodologies.

### 3.4 Mechanisms exploration underlying NIR pacing with TFP

**FIGURE 4:**
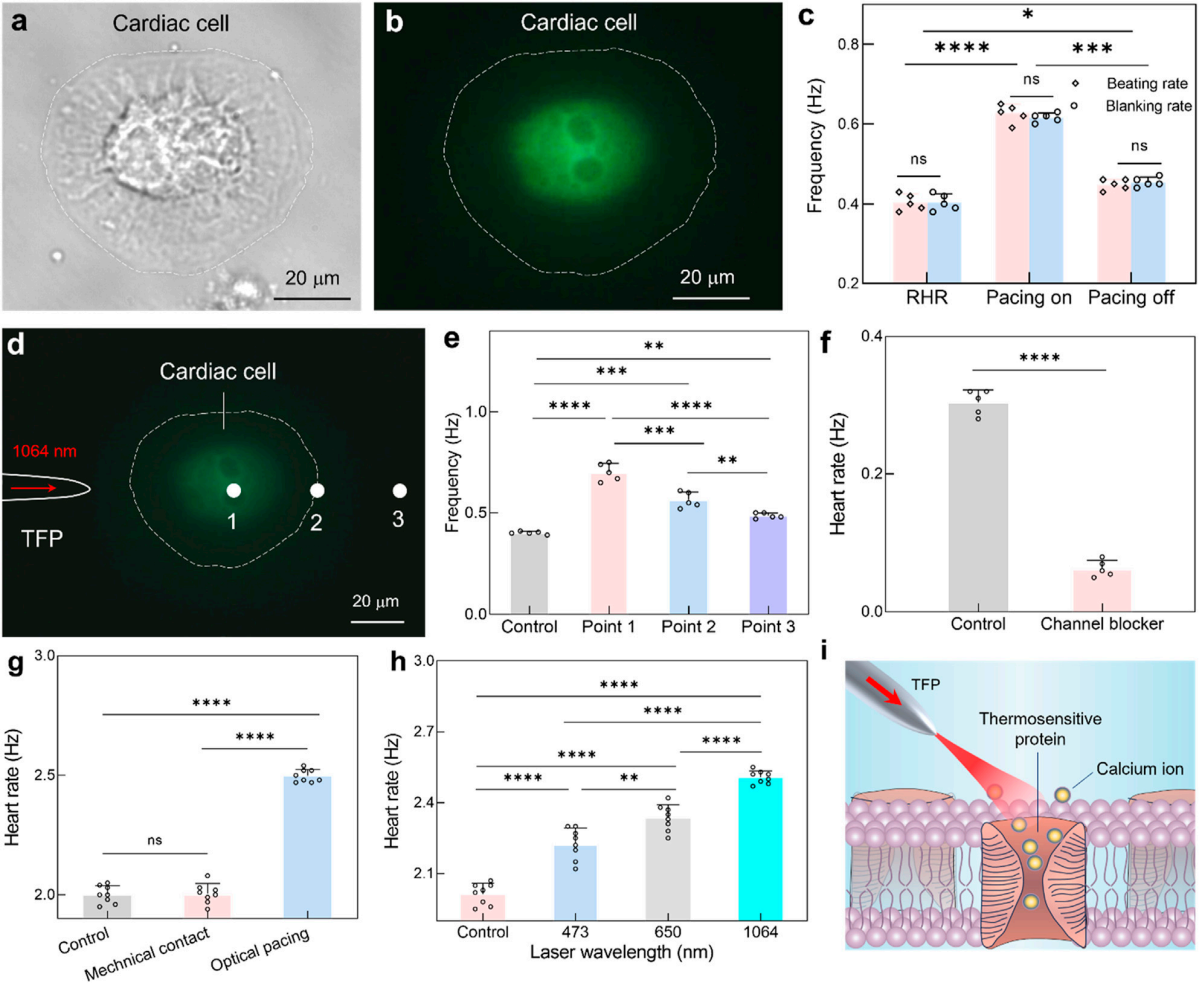
Mechanism exploration underlying NIR pacing with TFP. **(a,b)** Brightfield micrograph **(a)** and calcium fluorescence imaging **(b)** of the cardiac cell. **(c)** Quantitative correlation between cardiomyocyte beating frequency and calcium transient rate before and during optical pacing. Results are presented as mean ± SD (*n* = 5 cells). Statistical comparison was analyzed by two-way ANOVA with Bonferroni correction. ns, not significant. ^*^ *P* < 0.05; ^***^ *P* < 0.001; ^****^ *P* < 0.0001. **(d)** Calcium fluorescence imaging during optical stimulation at three different locations via TFP. **(e)** The calculated heart rate as a function of optical stimulation position. Results are presented as mean ± SD (*n* = 5 cells). Statistical comparison was analyzed by one-way ANOVA with Bonferroni correction. ns, not significant. ^**^ *P* < 0.01; ^***^ *P* < 0.001; ^****^ *P* < 0.0001. **(f)** Comparative analysis of cardiac cell beating rhythm for the control group and channel blocker group. Data are reported as mean ± SD (*n* = 5 cells). Statistical comparison was analyzed by two-tailed *t*-test with Welch correction, assuming Gaussian distribution. ^****^ *P* < 0.0001. **(g)** The calculated heart rate under direct mechanical contact and optical pacing. Results are presented as mean ± SD (*n* = 8 larval zebrafish). Statistical comparison was analyzed by one-way ANOVA with Bonferroni correction. ns, no significant; ^****^ *P* < 0.0001. **(h)** The calculated heart rate under optical pacing while using different laser wavelength. Results are presented as mean ± SD (*n* = 8 larval zebrafish). Statistical comparison was analyzed by one-way ANOVA with Bonferroni correction. ns, no significant; ^**^ *P* < 0.01; ^****^ *P* < 0.0001. **(i)** Schematic illustrating the opto-thermal mechanism underlying near-infrared cardiac pacing.

Following the demonstration of targeted optical pacing in zebrafish hearts, the physiological mechanisms underlying NIR optical pacing with TFP were investigated to optimize pacing flexibility through integrated approaches. For this goal, real-time calcium imaging was implemented concurrently with NIR optical stimulation of cardiomyocytes. As shown in Figures 4a,b, upon optical stimulation via the TFP, an immediate transient enhancement in calcium fluorescence intensity was observed in cardiomyocytes. This response was attributed to the opening of membrane calcium channels by NIR irradiation, thus triggering an enhanced calcium influx (Li et al., 2023). Afterwards, the beat rhythm of cardiac cell was observed to accelerate in accordance (Supplementary Figure S2; Supplementary Movie S2). Furthermore, the relationship between calcium transient frequency and beating rhythm of cardiac cells was quantitatively analyzed (Figure 4c). The beating rhythm of cardiomyocytes was found to exactly match the calcium scintillation rate both before and during stimulation. This temporal correlation validated that NIR optical pacing operates through calcium channels modulation, with subsequent calcium influx mediating the observed acceleration effect.

However, there are abundant transmembrane proteins distributed onto the cardiomyocyte membranes, including thermos-triggered, light-gated, and mechanosensitive proteins (Caporizzo and Prosser, 2022). Thus, it should be explored for the specific protein which was responsible for mediating NIR optical pacing. Leveraging the high spatial resolution of TFP, targeted NIR irradiation was applied to three subcellular locations (Figure 4d), *i.e.*, position 1, 2 and 3 for the cell body, cell periphery and extracellular space adjacent to the cell, respectively. Notably, the above positions are explicitly spaced at 32 μm intervals, *i.e.*, about 5-fold of the spot diameter, thus allowing discrimination between direct cardiomyocyte irradiation and extracellular effects. Meanwhile, the frequency of cardiomyocyte beating and calcium transients imaging were recorded simultaneously during optical stimulation (Supplementary Figure S3). As quantified in Figure 4e, the resting beating frequency was measured to be 0.4 Hz. The NIR light irradiated at position 1 elicited the beating frequency increase to 0.7 Hz. In comparison, optical stimulation at position 2 yielded a reduced response (0.55 Hz), likely attributable to decreased effective pacing area. Crucially, the heartbeat will also accelerate even when the light was irradiated onto position 3 (0.48 Hz). Due to the spatial separation between NIR light and cardiac cell, this location precludes direct activation of light-gated or mechanosensitive protein, and thus, the observed photomodulation should be attributed to light-induced localized temperature elevation for the dynamic opening of thermosensitive channels.

While direct measurement of localized temperature elevation would provide compelling evidence for the photothermal mechanism, implementing micro-scale thermometry in living zebrafish embryos without perturbing their physiological environment remains technically challenging. As an alternative approach, a well-established photothermal model was employed that confirms near-infrared laser irradiation at 1064 nm produces linear temperature increases proportional to optical power density, with a characterized coefficient of Δ*T*/*P*_density_ = 2.61 °C ± 0.4 °C⋅μm^2^/mW (Ebert et al., 2007). Thus, for the given threshold power density (*i.e.*, 0.53 mW/μm^2^), a desired temperature elevation could be induced with a magnitude of Δ*T* = 1.38 °C ± 0.2 °C. Meanwhile, the volume of heated medium was estimated to interpret the hypothetical optothermal mechanism comprehensively. For continuous-wave NIR stimulation, heat diffusion occurs radially from the beam center, forming a hemispherical volume. Thus, the heated volume *V* could be estimated as follows: *V* = 2/3×π × δ^3^, where *δ* = $\sqrt{\alpha \tau }$ was the characteristic diffusion length, *α* = 1.4 × 10^−7^ m^2^/s was thermal diffusivity of cell culture and *τ* was the stimulation duration. Take *τ* = 30 s, for example, the *δ* and *V* was measured to be 65 μm and 0.38 nL, respectively. Thus, the 30-s optical irradiation at Position 3 can achieve an efficient thermal profile with a radius of 65 um, which extends to the cardiac cell region and affects the cell beating through thermal conduction. Moreover, multiple studies confirm cardiomyocytes express transient receptor potential vanilloid 1 (TRPV1), which act as temperature-sensitive calcium gates and modulate cardiac excitability (Gorbunov et al., 2019; Yoshie et al., 2020). Under infrared radiation. these thermos-TRP channels could experience conformational changes via rapid photothermal effects, thus triggering the inflow of Ca^2+^ influx and inducing enhanced cardiac contraction dynamics (Wu P. et al., 2024). Therefore, when irradiating at point 3 with continuous laser light for 30 s, the heated solution transfers thermal energy to the cellular vicinity via thermal conduction, thus elevating local temperature at the cell membrane and activating thermosensitive ion channels to modulate myocardial contraction rhythm. To demonstrate potential involvement of thermosensitive channel, an additional pharmacological inhibition experiment was performed by using the thermosensitive channel blocker, *i.e.*, Capsazepine with a concentration of 0.1 μmol/L. As shown in Figure 4f, the administration of channel blocker resulted in a significant attenuation of the beating rate increase elicited by optical stimulation, which supports the conclusion that thermosensitive channels are actively involved in mediating the cardiac cell response to localized photothermal stimulation. Furthermore, the pacing effect was also characterized by adjusting the buffer temperature, while systemic thermal elevation might influence several physiological mechanisms (Souza et al., 2025; Toni et al., 2023) and fail to achieve tissue-specific stimulation of targeted myocardial regions.

In addition to the optothermal mechanism, the potential intervention was also explored for the mechanical simulation and photochemical stimulation. To assess mechanical stimulation from fiber probe placement, the TFP was positioned identically near the larval zebrafish (as in the main experiments) but with the laser turned off (Figure 4g). This setup ensured that any mechanical interaction from the probe itself (*e.g.*, physical contact or fluid disturbance) was replicated without optical energy delivery. The results exhibited no significant change in heart rate, which confirms that mechanical contact or proximity of the TFP does not elicit cardiac pacing. Moreover, the blue light (473 nm) and red light (650 nm) were selected as comparators for the primary NIR light (1064 nm) while at identical power (*i.e.*, *P* = 60 mW). Notably, the heart rate was increased to 2.22 ± 0.07 Hz at 473 nm stimulation, but with significant variability due to tissue scattering (Figure 4h). For the case of 650 nm stimulation, the heart rate was increased to 2.33 ± 0.05 Hz, showing improved efficacy over blue light but still with limited modulation flexibility. In contrast, the 1064-nm stimulation induced a maximum heart rate of 2.5 ± 0.02 Hz, demonstrating the most robust and reproducible pacing effect, which is consistent with its reduced scattering and deeper penetration in biological tissues, thus enabling more efficient energy delivery to cardiomyocytes.

Based on these above results, the primary mechanism was established for the NIR pacing with TFP: During NIR optical stimulation with TFP, photothermal effects from tissue absorption activate thermosensitive calcium channels (Figure 4i), after which subsequent calcium influx triggers rapid membrane depolarization and accelerates cardiomyocyte contraction. While potential contributions from light-gated proteins require further investigation, thermosensitive channel activation could be identified as the dominant pathway under these observed NIR pacing by using TFP. Nevertheless, the temperature elevation may also modulate the protein channels by other biophysical mechanisms concurrently, such as changing membrane capacitance (Pinto et al., 2022), inducing partial dissociation of F-actin (Burbaum et al., 2021) and accelerating sarcomeric oscillation (Sander and Zhu, 2024; Shintani, 2022), which remains a critical area for further research to decouple the primary effect from these secondary modulations.

## 4 Discussion

This study establishes TFPs as a powerful tool for precise cardiac pacing by leveraging their unique long-range focusing capability to achieve sustained micron-scale spatial resolution while at physiologically relevant working distances. Notably, the critical issue of fabrication reproducibility and modulation uniformity should be explored for the potential biomedical application. Although primary data were collected using a single characterized TFP to ensure experimental consistency, validation experiments with multiple probes confirmed minimal inter-probe variability (Supplementary Figure S4). In addition, the safety concerns should be addressed due to the unavoidable tissue absorption and potential heating under a maximum applied power density of 2.1 mW/μm^2^. For this purpose, a series of additional experiments have been performed to examine the potential biological damage onto the zebrafish embryo by the laser beam. Under employed optical power levels for cardiac rhythm modulation, comparative analysis of optical pacing versus unmanipulated zebrafish embryos revealed equivalent survival rates (Supplementary Figure S5a), *i.e.*, 96% and 94%, respectively. Meanwhile, quantitative assessments also demonstrated no significant differences in resting heart rate (Supplementary Figure S5b), thereby reconfirming the high biocompatibility of the proposed optical methodology for further *in vivo* applications.

Crucially, substantial efforts have been made to develop the powerful pacing strategy for the desired cardiac pacing *in vivo*. Among these, optogenetic approaches achieve exceptional temporal precision and cell-type specificity through genetic targeting of light-sensitive ion channels, making it particularly valuable for precise cardiac pacing studies in transgenic models (Entcheva and Kay, 2021; Langen et al., 2025). However, the required viral transfection or genetic modification may alter native electrophysiology and introduce immunogenicity concerns, thereby limiting its immediate clinical translatability. Photoacoustic techniques, which combine optical excitation with ultrasonic energy transfer, offer superior tissue penetration depth compared to purely optical methods, thereby showing promise for imaging and stimulating deeper cardiac structures (Du et al., 2024; Zhou et al., 2025). The trade-off, however, is typically lower spatial resolution, which may restrict its application in scenarios requiring cellular-scale precision. In addition, electrothermal simulation utilizes resistive heating elements to achieve thermal stimulation of cardiac tissue (Wang Z. et al., 2025). Unfortunately, these approaches typically involve implanted electrodes that may cause electrochemical tissue damage and are susceptible to electromagnetic interference. Compared to these potential modulation strategies, the proposed TFP platform offers distinct advantages, including non-genetic operation that preserves native cellular electrophysiology, cellular-resolution spatiotemporal mapping of discrete pathways and dynamic parameter modulation for simulating pathological conduction patterns. Its MRI compatibility permits simultaneous pacing and functional imaging, which remains impossible with electronic devices due to electromagnetic interference. For potential clinical translation, a closed-loop optical pacemaker could be developed by integrating real-time ECG monitoring, automatic feedback control, and TFP-based focusing enhancement for subtype-specific pacing. The platform’s minimal invasive footprint (<10 μm probe diameter) and absence of electrochemical reactions address key failure modes of conventional pacemakers. Particularly, by organic integration with fiber-optic endoscopy, this technology overcomes the depth constraints of conventional optical strategies, enabling programmable optical pacing within deep-seated cardiac regions while achieving on-demand modulation of pacing sites. Despite these advances, limitations require addressing. Prolonged stimulation might increase local temperature which necessitates thermal monitoring algorithms for chronic applications. Meanwhile, while effective in zebrafish embryos, it still faces great challenges towards the clinical translation due to the enhanced optical scattering in adult mammalian hearts. Moreover, differential thermosensitive channel expression across cardiomyocyte subtypes may cause different pacing thresholds in complex tissues, and scaling probe dimensions for human hearts while maintaining micron-scale precision requires novel fabrication approaches. Future research should prioritize long-term biocompatibility studies, multiplexed probe arrays for coordinated large-scale pacing, machine learning optimization of stimulation parameters, and large-animal validation in porcine arrhythmia models.

## 5 Conclusion

In conclusion, this work establishes a TFP-based nongenetic platform for non-contact and high-precision near-infrared optical pacing within living zebrafish embryos. By leveraging the TFP’s excellent long-range focusing capability, micro-scale spatial precision was achieved in targeted cardiomyocyte stimulation, enabling localized optical excitation with cellular resolution. Systematic quantification revealed critical dependencies of pacing efficacy on developmental stage, demonstrating progressive sensitivity reduction during early cardiogenesis (3-6 dpf) followed by stabilization upon cardiac maturation (>6 dpf), and anatomical location, with sinoatrial tissue exhibiting 1.7-fold greater responsiveness than ventricular myocardium. Mechanistically, optical pacing operates primarily through photothermal activation of thermosensitive calcium channels, triggering Ca^2+^ influx and subsequent membrane depolarization. The tapered fiber probe platform presents distinct advantages over conventional electrical pacing, including superior spatial resolution, inherent MRI compatibility due to absence of metallic components, and dynamic parameter tunability via optical power modulation. While its application to human pacemakers requires extensive future validation in mammalian models and clinical settings, the proposed TFP-based approach might offer a potential tool to explore optical pacing mechanisms and perform basic arrhythmia research *in vivo*.

## Data Availability

The original contributions presented in the study are included in the article/Supplementary Material, further inquiries can be directed to the corresponding authors.
