# Supplementary material for: Tapered fiber probe-based optical cardiac pacemaker
Source: Front Bioeng Biotechnol. 2025 Nov 11;13:1675219. doi: 10.3389/fbioe.2025.1675219 (PMC12643975; doi:10.3389/fbioe.2025.1675219)
Supplement: Supplementary file 1 [file Supplementaryfile1.docx]

Supplementary Material

Tapered fiber probe-based optical cardiac pacemaker

Yanzheng Xie^1*^, Xiaoshuai Liu^2*^

^1^ Jiangsu Vocational College of Medicine, Yancheng, 224005, China

^2^ School of Physics and Materials Science, Guangzhou University, Guangzhou 510006, China.

*** Correspondence:** [xieyzheng@outlook.com](mailto:xieyzheng@outlook.com) (Y. X.) or [lxshuai@gzhu.edu.cn](mailto:lxshuai@gzhu.edu.cn) (X. L.).

**Table of Contents**

**Figure S1**. Experimental measurements of the light spot.

**Figure S2**. Extracted traces of calcium waves and contraction analysis before and after optical pacing.

**Figure S3**. Extracted traces of cardiac cell beating as a function of optical stimulation position.

**Figure S4**. Performance validation of TFP fabrication reproducibility and cardiac modulation efficiency.

**Figure S5.** Biosafety characterization of optical pacing.

# Experimental measurements of the light spot

The light spot was measured using the knife-edge technique, which employs a sharp edge to scan the beam while a photodiode detects intensity variations during scanning. Crucially, this characterization was performed under identical conditions mirroring our experimental setup, *i.e.*, a 50 μm separation distance between the fiber probe tip and cardiac tissue surface, replicating the *in vivo* non-contact stimulation paradigm. As indicated in **Figure S1**, the measured beam diameter at this working distance exhibited a full-width half-maximum (FWHM) of 6 μm. Nevertheless, it should be noted that the actual spot radius reaching subsurface myocardial tissue will exceed 6 μm due to unavoidable light-tissue interactions, including scattering and divergence effects within the cardiac microenvironment.


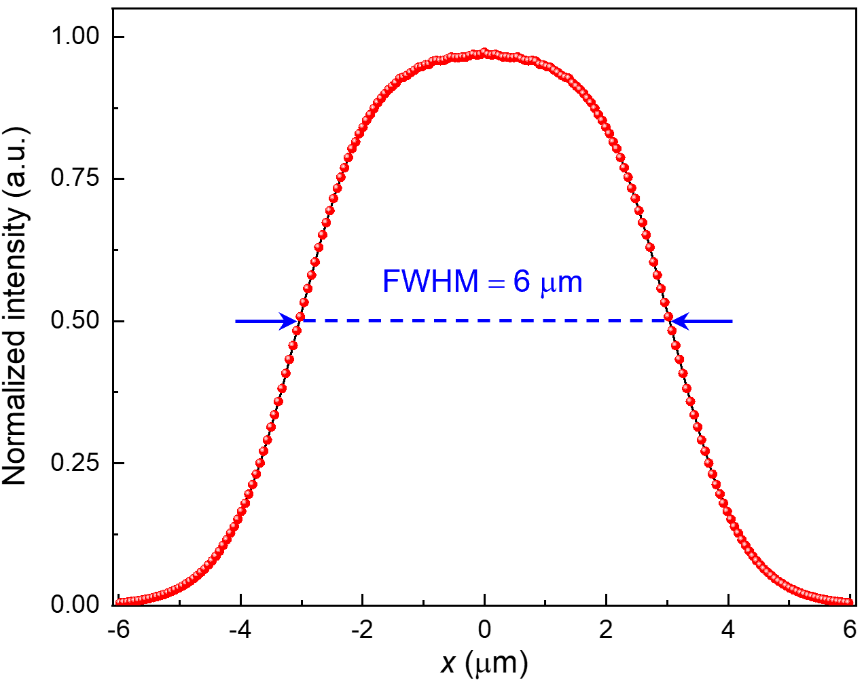


**Figure S1**. Experimental measurements of the light spot.

# Extracted traces of calcium waves and contraction analysis

The representative traces of cardiac cell beating (top panel) and calcium transient waves (bottom panel) were calculated during TFP-mediated optical pacing (**Figure S2**). Under pre-stimulation conditions, cardiomyocytes exhibited stable spontaneous contractions at 0.4 Hz, with precisely correlated calcium oscillation frequency of 0.4 Hz, confirming intrinsic excitation–contraction coupling. Upon initiation of optical stimulation via the TFP, both contraction frequency and calcium oscillation frequency were increased synchronously to 0.6 Hz, indicating enhanced cellular excitability. Following cessation of laser irradiation, both parameters returned to baseline levels (0.4 Hz) within seconds, demonstrating the reversible nature of photothermal pacing. These parallel recordings validate the tight coupling between calcium handling and mechanical function throughout the optical stimulation protocol.


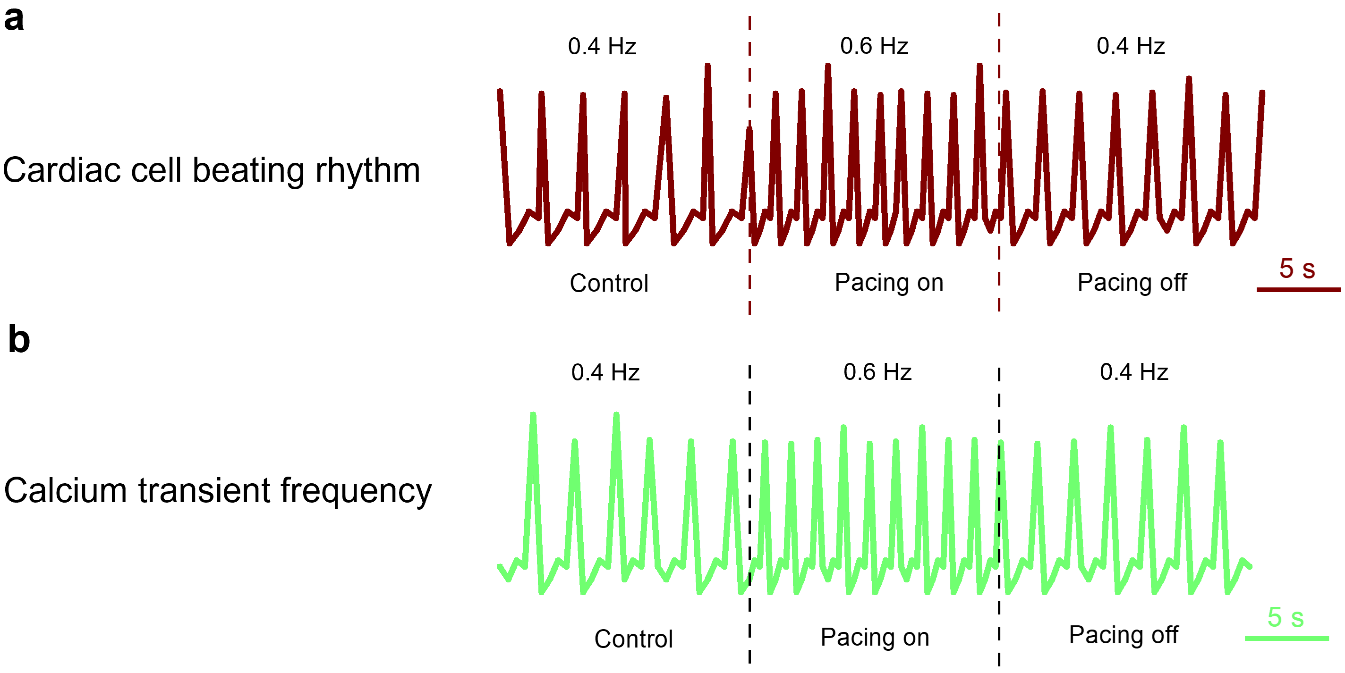


**Figure S2**. Extracted traces of calcium waves (a) and contraction analysis (b) before and after optical pacing.

# Extracted traces of cardiac cell beating

To elucidate the physiological mechanism of TFP-mediated cardiac rhythm modulation, we systematically delivered optical stimulation at three distinct locations: extracellular space (32 μm from cell membrane, Position 3), cell periphery (Position 2), and directly on the cell body (Position 1), while recording contraction dynamics. Quantitative analysis revealed a spatially graded response, with stimulation at Position 3 elevating contraction frequency to 0.48 Hz, Position 2 to 0.55 Hz, and Position 1 achieving maximal efficacy at 0.70 Hz, compared to the baseline of 0.4 Hz. The results underscore the precision of tapered fiber probe technology in achieving location-dependent stimulation while providing critical insights into the photothermal activation pathway governing cardiac pacing.


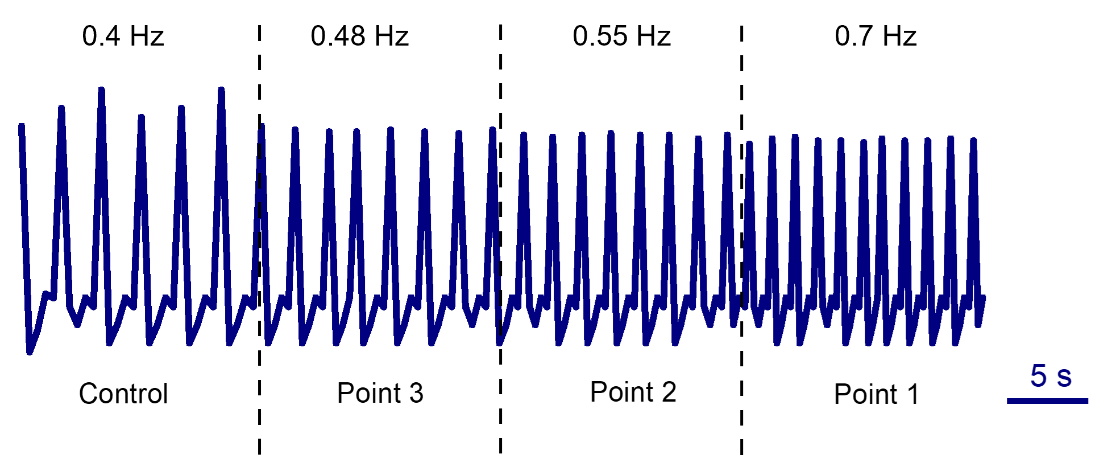


**Figure S3**. Extracted traces of cardiac cell beating as a function of optical stimulation position.

4. **Performance validation of TFP fabrication reproducibility and cardiac modulation efficiency**

For the issue of reproducibility, the used flame-heating technique yields >90% geometric consistency across independently fabricated probes, thus confirming high reproducibility to minimizes inter-probe variability (**Figure S4**a). Furthermore, the validation experiments was performed by using the four additional probes from the same fabrication batch (**Figure S4**b), which show negligible differences in pacing efficiency with a same average heart rate increase of 0.52 Hz at the same power density of *P*_density_ = 2.12 mW/μm^2^.


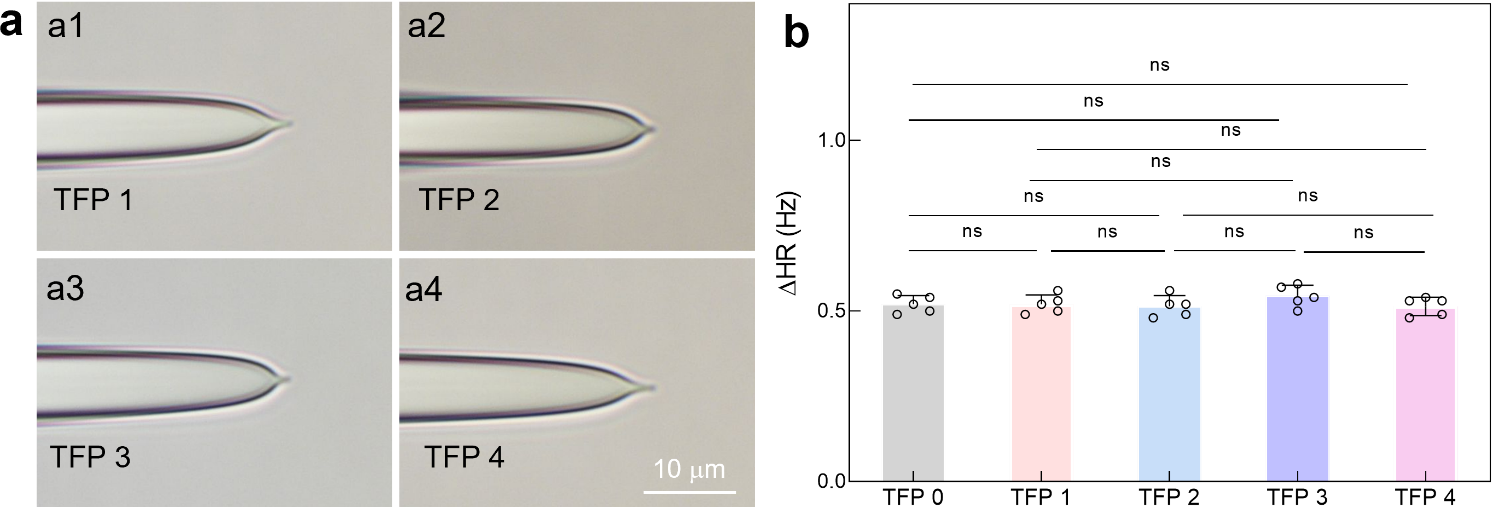


**Figure S4**. (**a**) Optical micrographs of the fabricated fiber probe with similar shapes based on the flame-heating technique. (**b**) The calculated heart rate modulation by using the above four TFPs (TFP 0 represents the originally used fiber probe). Results are presented as mean ± SD (*n* = 5). Statistical comparison was analyzed one-way ANOVA with Bonferroni correction. ns, not significant.

**5.** **Biosafety characterization of optical pacing**

To comprehensively assess the biosafety associated with optical pacing, the embryonic survival rates and resting heart rhythms were systematically evaluated in both pacing group and control group. As illustrated in **Figure S5**a, the survival rate of zebrafish embryos subjected to optical pacing (96%) was highly comparable to that of control group (94%), indicating no significant adverse effects on viability. Furthermore, quantitative analysis of resting heart rates revealed nearly identical values (**Figure S5**b), *i.e.*, 2.0 Hz for the pacing group versus 2.05 Hz for the control group, with no statistically significant difference.


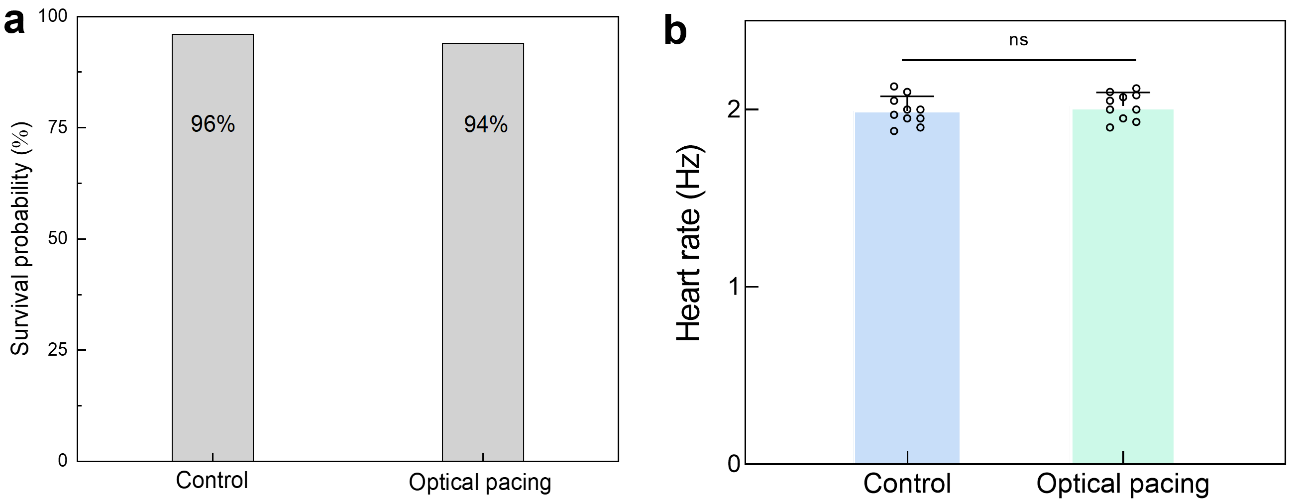


**Figure S5.** Comparative analysis of embryonic survival rate (a) and heart rate (b) for the control group and optically pacing group. Data are reported as mean ± SD (*n* = 10 larval zebrafish). Statistical comparison was analyzed by two-tailed *t* test with Welch correction, assuming Gaussian distribution. ns, no significant.
